# Supplementary material for: Direct observation of exciton–exciton interactions
Source: Nat Commun. 2018 Jun 25;9:2466. doi: 10.1038/s41467-018-04884-4 (PMC6018121; doi:10.1038/s41467-018-04884-4)
Supplement: Supplementary file 1 — Supplementary Information [file 41467_2018_4884_MOESM1_ESM.pdf]

SUPPLEMENTARY INFORMATION FOR

Direct observation of exciton–exciton interactions

Dostál et. al.

Supplementary Note 1: Further Properties of the EEI Signal

Non-Rephasing and Quantum Beating Signals of Annihilation Processes

The non-rephasing EEI signal characterized by the $2\mathbf{k}_1 - 2\mathbf{k}_2 + \mathbf{k}_3$ phase-matching relation yields a time evolution identical to its rephasing counterpart. Just as in absorptive 2D spectroscopy, for each rephasing double-sided Feynman diagram there is a corresponding non-rephasing one. This is demonstrated in Supplementary Figure 1 for the case of a pair of two-level systems with the same idealized annihilation channel as presented in the main paper. The detected EEI2D signal thus consists of absorptive-type (rephasing + non-rephasing) line shapes.

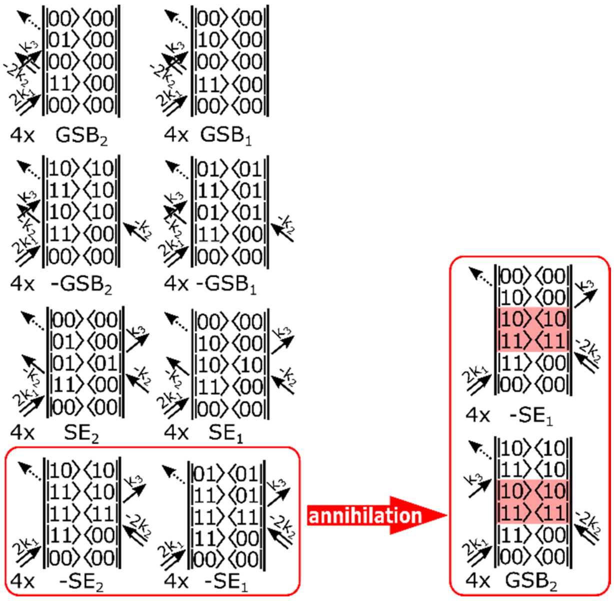

Supplementary Figure 1: Double-sided Feynman diagrams of the non-oscillatory non-rephasing EEI signal of the model system of exciton annihilation. All diagrams are exact counterparts of the rephasing diagrams shown in Figure 1, together forming "absorptive" line shapes. For details on notation see the caption of Figure 1.

Figure 1 and Supplementary Figure 1 contain those diagrams in which the system is in a population state (i.e., a diagonal element in the density matrix) during the nominal population time. In addition, it is possible to construct diagrams in which both constituting subsystems are simultaneously present in coherent states (Supplementary Figure 2). However, each type of such an oscillatory diagram is present twice with mutually opposite signs, which leads to a perfect cancelation of quantum beating signals.

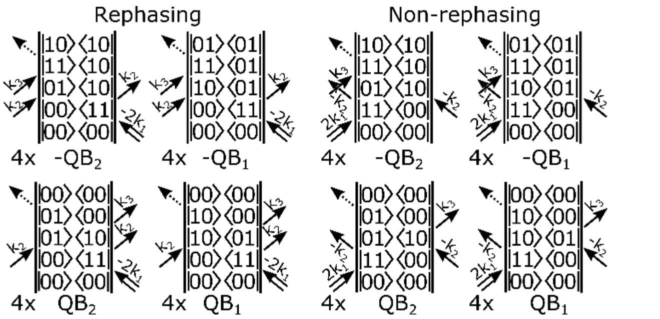

Supplementary Figure 2: Double-sided Feynman diagrams of the oscillatory EEI signal of the model system. All diagrams perfectly compensate each other.

Relation between Fifth-Order Signals

The interactions between excitons are responsible for various types of fifth-order three-pulse signals. In this work we investigate in detail signals characterized by the $-2\mathbf{k}_1 + 2\mathbf{k}_2 + \mathbf{k}_3$ and $2\mathbf{k}_1 - 2\mathbf{k}_2 + \mathbf{k}_3$ phase-matching relations that lead to EEI2D spectra. For the purpose of this subsection we will denote the signals by their position of appearance in the 2D spectra as 2ω-R and 2ω-NR for the rephasing and nonrephasing contributions, respectively. Other fifth-order signals,

$$-\mathbf{k}_1 + (\mathbf{k}_1 - \mathbf{k}_1) + \mathbf{k}_2 + \mathbf{k}_3,$$
$$-\mathbf{k}_1 + \mathbf{k}_2 + (\mathbf{k}_2 - \mathbf{k}_2) + \mathbf{k}_3,$$
$$\mathbf{k}_1 + (\mathbf{k}_1 - \mathbf{k}_1) - \mathbf{k}_2 + \mathbf{k}_3,$$
and $\mathbf{k}_1 - \mathbf{k}_2 + (\mathbf{k}_2 - \mathbf{k}_2) + \mathbf{k}_3$,

overlap spectrally with the conventional absorptive 2D spectrum and are responsible for observing the bi-exciton effects there. We abbreviate these signals as 1ω-1-R, 1ω-2-R, 1ω-1-NR, and 1ω-2-NR, respectively. In addition, signals of the type $\pm(\mathbf{k}_1 - \mathbf{k}_1) \pm (\mathbf{k}_2 - \mathbf{k}_2) + \mathbf{k}_3$ may appear near the origin of the excitation axis of the 2D spectrum (denoted as 0ω). However, in our experiments 0ω signals are entirely suppressed by the phase-cycling scheme.

The common feature of all the fifth-order signals is that the first four interactions with the electric field happen during two laser pulses only. There are multiple ways for how the identical sequence of four interactions can be distributed within two groups, therefore the same piece of information (represented by the same general fifth-order diagram) appears multiple times among 0ω, 1ω, and 2ω signals.

Unlike 2ω signals, the 0ω and 1ω signals are not fully specific to EEI, since they can be constructed even for a two-level system. Here they describe the signal saturation with increasing excitation power. For the non-interacting pair of such systems the number of saturation diagrams doubles. In addition, there appears the self-cancelling set of diagrams specific to EEI analogous to diagrams of 2ω signals.

As any diagram that ends up in the two-exciton state (|11>) during the population time is part of this EEI-specific subset the relative abundance of the EEI-specific contribution in the various fifth-order signals can be determined by counting the number of such diagrams for each fifth-order signal.

In Supplementary Figure 3 we show all six possible interaction sequences that result in the presence of two excitons in the system during the population time. We see that all of them can be used for construction of 1ω-1- and 1ω-2-type signals, depending on whether the interactions are distributed between the two laser pulses in 3+1 or 1+3 fashion, respectively. However, only two of the sequences lead to 2ω signals. Thus, there are six times more such diagrams present in 1ω signals than in 2ω signals, and therefore any effects connected with EEI will be observed 6-times stronger in absorptive 2D spectra than in EEI2D spectra.

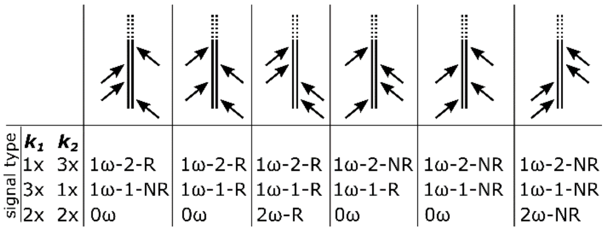

signal type k_1	k_2						
1x	3x	1ω-2-R	1ω-2-R	1ω-2-R	1ω-2-NR	1ω-2-NR	1ω-2-NR
3x	1x	1ω-1-NR	1ω-1-R	1ω-1-R	1ω-1-R	1ω-1-NR	1ω-1-NR
2x	2x	0ω	0ω	2ω-R	0ω	0ω	2ω-NR

Supplementary Figure 3: All possible classes of double-sided Feynman diagrams that result in a two-exciton state after four interactions with the electric field (top) and the character of the fifth-order signal caused by a given photon sequence of the two pulses $\mathbf{k}_1$ *and* $\mathbf{k}_2$ *(bottom).*

The individual contributions in absorptive 2D and EEI2D spectra are distributed along the excitation axis differently. Therefore, there is no easy way how to reconstruct the interaction-free absorptive 2D spectra from a combination of (high-excitation-intensity) absorptive 2D and EEI2D spectra. However, if the absorptive 2D and EEI2D spectra are integrated along the excitation axis it is possible to reconstruct the interaction-free transient absorption signal by subtracting six times the latter integrated spectrum from the former one. Analogously, by additional integration along the detection axis it is possible to reconstruct an interaction-free integrated transient absorption signal.

In case the actual experimental implementation of the data acquisition influences the relative intensities of the absorptive and EEI part of the spectra the correcting factor might change. In our case the employed phase cycling scheme emphasizes the EEI2D signal twice (see below), therefore the reconstruction of the interaction-free kinetics (as presented in Figure 6) is performed by subtracting three times the integrated EEI2D signal from the integrated absorptive 2D signal.

Supplementary Note 2: Quantitative Analysis of Experimental Data

In order to extract quantitative physical information from the experimental data we set up in this section a model that describes the exciton dynamics in the MEH-PBI aggregates including the effect of annihilation, the two-exciton state, and saturation.

Assumptions

We assume that the MEH-PBI aggregates can be well described as an infinitely long linear chain of identical two-level systems ("sites"), with an electronic coupling between adjacent sides of J. Disorder effects localize the exciton wave function to spread over δ sites, which we will call a domain in the following. For such an aggregate the energy level structure can be approximated in terms of Frenkel exciton theory.[1] Any other influences of the disorder except the exciton (de)localization are not taken into account in our model. We assume that the exciton hops randomly between domains and that this can be described by a diffusion equation as presented previously.[2–8] For the current model system and the applied excitation energies, we assume that exciton–exciton annihilation and the direct excitation of the two-exciton state are the only forms of interaction between pairs of excitons.

Transient Dipole Moment Structure

The energy structure of an exciton delocalized over δ sites consist of 2^{δ} states that are grouped in δ+1 groups,

each containing $N_\sigma \equiv \binom{\delta}{\sigma}$ states, where the notation in brackets refers to the binomial coefficient. Here $\sigma \epsilon \{0, 1, \dots N\}$ corresponds, consecutively, to the ground state, one-, two-, three-, and higher-exciton bands. In order to describe the transitions, we need to estimate relative values of the transition dipole moments allowing optical transitions between the consecutive bands.

Instead of characterizing transition dipole moments between each pair of states of each band (exact formulas can be partly found elsewhere[9,10]) we will approximately represent the entire band by a single totally symmetric wave function

$$\Psi_\sigma = \frac{1}{\sqrt{N_\sigma}} \sum_{i=0}^{N_\sigma} |\varphi_{\sigma i}\rangle, \quad (1)$$

where $|\varphi_{\sigma i}\rangle$ is the site basis of the σ exciton state indexed by i. Members of $|\varphi_{\sigma i}\rangle$ are constructed to contain all possible combinations of σ sites excited and δ-σ unexcited. The transition dipole moment value between two consecutive states Ψ_σ and $\Psi_{\sigma+1}$ thus represents an effective transition strength between the two bands and it can be expressed as

$$\left|\mu_{\sigma,(\sigma+1)}\right|^2 \equiv \left|\langle \Psi_\sigma | \mu | \Psi_{(\sigma+1)} \rangle\right|^2 = (\delta - \sigma)(\sigma + 1), \quad (2)$$

where

$$\mu = \sum_{l=1}^{\delta} \mu_l \quad (3)$$

is the total dipole moment operator constructed as the (direct) sum of the transition dipole moment operators μ_l of the constituting sites. Since we assume all sites identical and the model does not depend on the absolute size of the dipole moment we can set $|\mu_l| = 1$ for all l.

In our model we are especially interested in explicit values for $|\mu_{01}|^2 = \delta$, $|\mu_{12}|^2 = 2(\delta\text{-}1)$ and $|\mu_{23}|^2 = 3(\delta\text{-}2)$. Note that for $\delta > 2$, $|\mu_{12}|^2 > |\mu_{01}|^2$. Therefore, a singly excited domain is more prone to further excitation compared to a domain being in the ground state.

Initial Exciton Distribution

The intensity of the direct double-excitation component in the EEI2D signal scales with the probability of exciting the same aggregate domain twice. In contrast, the annihilation component of the EEI signal depends on two singly excited domains located within the average exciton diffusion length. Therefore, in order to get the relative intensity of both contributions in the EEI2D spectra we need to estimate the probabilities of single and double excitations of individual aggregate domains.

As a starting point we take the Poisson distribution and expand it to second order in a Taylor series. Thus we can quantify the fraction of non-, singly- and doubly-excited domains as

$$P'_0 = e^{-\lambda} \approx 1 - \lambda + \tfrac{1}{2}\lambda^2, \quad (4a)$$

$$P'_1 = \lambda e^{-\lambda} \approx \lambda - \lambda^2, \quad (4b)$$

$$P'_2 = \tfrac{1}{2}\lambda^2 e^{-\lambda} \approx \tfrac{1}{2}\lambda^2, \quad (4c)$$

respectively, where $\lambda = \delta\, n_{abs}/N$ is the average number of absorbed photons (n_{abs}) per single domain, with N indicating the number of molecules and N/δ the number of domains.

The Poisson distribution could be directly applied if the absorption events were fully uncorrelated. However, this is not valid for the aggregate since the capture of a photon by an already excited domain is $2(\delta\text{-}1)/\delta$ times more likely than by an unexcited one, which leads to a larger number of doubly excited domains and a smaller number of singly excited domains than predicted by the Poisson distribution. We will reflect this situation by scaling up P'_2 accordingly at the expense of P'_1, which leads to the following distribution:

$$P_0 = P'_0 = 1 - \lambda + \tfrac{1}{2}\lambda^2, \quad (5a)$$

$$P_1 = P'_1 - (P_2 - P'_2) = \lambda - \frac{3/2\delta - 1}{\delta}\lambda^2, \quad (5b)$$

$$P_2 = \frac{2(\delta-1)}{\delta} P'_2 = \frac{\delta-1}{\delta}\lambda^2. \quad (5c)$$

A minor inconvenient aspect of the distribution (5) is that its mean value $\langle P\rangle$ (i.e., the number of absorbed photons per domain) is not λ but increases to

$$\langle P\rangle = P_1 + 2P_2 = \lambda + \frac{1}{\delta}\left(\frac{\delta}{2} - 1\right)\lambda^2. \quad (6)$$

The physical reason is that the domain excitation increases the transition dipole moment to higher exciton states, which simplifies the harvesting of the second photon. An increase of the excitation power leads to a larger number of excited molecules that are capable of capturing more photons from the incident photon flux than would be expected from simply linearly scaling up

the number of captured photons determined at low-light conditions.

Taking this in mind we have to re-interpret the parameter λ in (5) as a linear extrapolation

$$\lambda = \xi\lambda_0 = \xi\frac{\delta n_0}{N} \quad (7)$$

from a value λ_0 determined at low excitation intensities. Parameter ξ is the linear scaling-up factor and n_0 is the number of photons absorbed at low-light conditions in which distributions (4) and (5) coincide. The average number of absorbed photons per single site (n_0/N) can be determined from the UV/vis absorption spectrum of the PBI sample and the respective overlap with the excitation laser spectrum as discussed below.

In our model we assume that any of the doubly excited domains quickly relaxes to its singly excited state and produces one "unit" of direct doubly excited signal and one exciton capable of further diffusion and annihilation. The number of excitons present in the sample capable of diffusion and annihilation is thus

$$n_{\mathrm{ann}}^{0} = \frac{N}{\delta}(P_1 + P_2) = \xi n_0\left(1 - \xi\frac{\delta}{2}\frac{n_0}{N}\right) \quad (8)$$

while the number of excitons responsible for the direct double excitation signal is

$$n_{\mathrm{dde}}^{0} = \frac{N}{\delta}P_2 = \xi^2(\delta - 1)\frac{n_0^2}{N}. \quad (9)$$

The total number of excitons initially present in the sample is

$$n_{\mathrm{total}}^{0} = \frac{N}{\delta}(P_1 + 2P_2) = \xi n_0\left(1 + \xi\left(\frac{\delta}{2} - 1\right)\frac{n_0}{N}\right). \quad (10)$$

Time Evolution of Integrated Absorptive 2D and EEI2D Signals

The decay of the exciton population due to diffusion-assisted annihilation can be described by the diffusion equation, which for the one-dimensional case can be solved analytically[6] as

$$n_{\mathrm{ann}}(t) = \frac{n_{\mathrm{ann}}^{0}\exp\left(-\frac{t}{\tau}\right)}{1 + \frac{n_{\mathrm{ann}}^{0}}{aN}\sqrt{2D\tau}\,\mathrm{erf}\left(\sqrt{\frac{t}{\tau}}\right)}, \quad (11)$$

where τ is the exciton lifetime due to relaxation to the ground state, a is the aggregate lattice constant, D is the exciton diffusion constant, and N is the number of illuminated sites.

The time evolution of the annihilation part of the integrated EEI2D signal is proportional to the accumulated number of annihilation events at a certain population time t, which can be calculated by the difference between the annihilation-free single-exponential decay and Eq. (11) as

$$S_{\mathrm{EEI}}^{\mathrm{ann}} = A\left(n_{\mathrm{ann}}^{0}\exp\left(-\frac{t}{\tau}\right) - n_{\mathrm{ann}}(t)\right)$$

$$= A\xi n_0\left(1 - \frac{1}{2}\xi\delta\frac{n_0}{N}\right)\frac{\exp\left(-\frac{t}{\tau}\right)\,\mathrm{erf}\left(\sqrt{\frac{t}{\tau}}\right)}{\frac{a}{\sqrt{2D\tau}\,\xi\frac{n_0}{N}\left(1-\frac{1}{2}\xi\delta\frac{n_0}{N}\right)} + \mathrm{erf}\left(\sqrt{\frac{t}{\tau}}\right)}, \quad (12)$$

where A is the proportionality constant between the number of excitons and the observed signal. The result can be simplified by introducing new parameters

$$\alpha = An_0, \quad (13)$$

$$\beta = \frac{a\frac{N}{n_0}}{\sqrt{2D\tau}}, \quad (14)$$

as

$$S_{\mathrm{EEI}}^{\mathrm{ann}} = \alpha\left(1 - \frac{1}{2}\delta\frac{n_0}{N}\xi\right)\xi\frac{\exp\left(-\frac{t}{\tau}\right)\,\mathrm{erf}\left(\sqrt{\frac{t}{\tau}}\right)}{\frac{\beta}{\left(1-\frac{1}{2}\delta\frac{n_0}{N}\xi\right)\xi} + \mathrm{erf}\left(\sqrt{\frac{t}{\tau}}\right)}. \quad (15)$$

The annihilation process can be thus described for any excitation intensity characterized by a varying parameter ξ and a fixed set of parameters α, β, δ, and τ.

The direct double-excitation part of the integrated EEI2D signal rises with exciton relaxation from the two-exciton state into the single-exciton state. We assume that this process can be well described with a single exponential decay characterized by the time constant τ_{dde}. The corresponding EEI signal can be again constructed as the difference between the real exciton decay and the ideal hypothetical interaction-free decay as

$$S_{\mathrm{EEI}}^{\mathrm{dde}} = An_{\mathrm{dde}}^{0}\left(\exp\left(-\frac{t}{\tau}\right) - \exp\left(-\frac{t}{\tau_{\mathrm{dde}}}\right)\right)$$

$$= \alpha(\delta - 1)\frac{n_0}{N}\xi^2\left(\exp\left(-\frac{t}{\tau}\right) - \exp\left(-\frac{t}{\tau_{\mathrm{dde}}}\right)\right). \quad (16)$$

If we assume that the two types of EEI signals do not influence each other (e.g., by different and uncorrelated diffusion properties of the exciton in the double-exciton state during its ultrafast lifetime), the time evolution of the total integrated EEI2D spectrum is simply given as sum of both contributions,

$$S_{\mathrm{EEI}} = w\left(S_{\mathrm{EEI}}^{\mathrm{ann}} + S_{\mathrm{EEI}}^{\mathrm{dde}}\right). \quad (17)$$

In Eq. (17) we explicitly added the factor *w* that weights the overall intensity of the EEI2D spectrum compared to its absorptive 2D counterpart due to the smaller number of Feynman diagrams as discussed above. Generally, *w* = 1/6; however, in our experiments where the EEI2D spectrum is enhanced twice by the used phase cycling scheme (see a section below for further information on phase cycling), this factor changes to *w* = 1/3.

The time evolution of the integrated absorptive 2D spectrum is given as

$$S_{\mathrm{abs}} = S_{\mathrm{free}} + \frac{1}{w} S_{\mathrm{EEI}}, \quad (18)$$

where S_{free} is the hypothetical interaction-free decay of all excitons present in the sample defined as

$$S_{\mathrm{free}} = -A n_{\mathrm{total}}^{0} \exp\left(-\frac{t}{\tau}\right)$$
$$= -\alpha\xi\left(1 + \xi\left(\frac{1}{2}\delta - 1\right)\frac{n_0}{N}\right)\exp\left(-\frac{t}{\tau}\right). \quad (19)$$

The model depends on five free parameters α, β, δ, τ and τ_{dde} which can be determined by a simultaneous fit of the integrated absorptive 2D and EEI2D spectra. However, in our experimental data the initial sub-100-fs time evolution is not reliably determined due to the uncompensated chirp in the probe light. Therefore, we excluded the first two data points (0 fs and 50 fs) from the fitting procedure and we set τ_{dde} to 50 fs, which is below our time resolution.

The role of the parameters of the model is the following. The exciton lifetime τ gives the overall time scale of the model curve. Similarly, the parameter α gives the scaling along the vertical axis. The parameter β has a straight-forward physical interpretation. It is the ratio between the average distance between individual excitons (aN/n_0) and the relative exciton diffusion length ($(2D\tau)^{1/2}$). Its inverse value β^{-1} thus gives the average number of excitons that each exciton can meet and annihilate with. The value of parameter β determines the shape of the diffusion-mediated annihilation part of the EEI signal via Eq. (15). With a higher value of β (i.e., fewer interacting excitons) the maximum of the EEI signal appears at later times. The exciton delocalization length δ scales the relative strengths of the two EEI processes in two ways. It scales up the probability for direct double excitation of the same domain, which becomes in the data apparent as an ultrafast rise. Simultaneously, it reduces the number of excitons capable of diffusion, which changes the shape and reduces the intensity of the annihilation curve as given by Eq. (15). The uniqueness of the fit is highly probable since each parameter is mostly responsible for one distinct feature of the fitted curve.

In real aggregates multiple additional factors that reach beyond our model might affect the probability of direct double excitation of a single domain. Firstly, the process might be allowed due to the presence of higher excited states of the monomeric constituents (sites),[11] not only by double-exciton states as we assumed here. Secondly, we have to consider that the transition dipole moments in between the bands of a single site were only approximated in our model. In addition, the exciton might decrease its delocalization length (localize) after its creation as observed for a small H-type aggregate consisting of a PBI.[12] The experimentally determined values of δ overestimate the real exciton delocalization length of the aggregate. Nevertheless, as can be seen in Figure 5 of the main text, the model presented above describes the data well within the given signal-to-noise ratio.

Supplementary Note 3: Further Experimental Details

MEH-PBI – High Excitation Intensity

The 2D spectra measured at a high excitation intensity of 34 nJ are shown in Supplementary Figure 4. The EEI2D part of the spectrum is stronger compared to the spectra taken at lower excitation intensities (Figure 4). In addition, the EEI2D signal is present from the very beginning due to the direct double-excitation process.

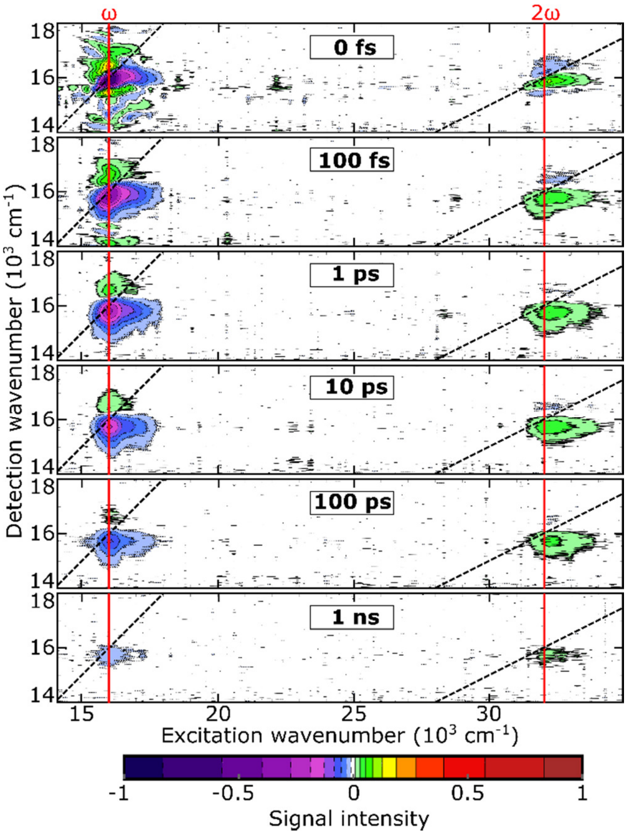

Supplementary Figure 4: EEI2D spectra (real part) of MEH-PBI aggregates at various population times for an excitation energy of 34 nJ. Contours are drawn at fractions of 0.01, 0.03, 0.05, 0.08, 0.13, 0.19, 0.27, 0.4, 0.57, and 0.83 of the maximal signal amplitude.

Nile Blue – Annihilation-Free Sample

As a control sample an ethanol solution of Nile blue of OD = 0.3 at 628 nm was used. This laser dye was chosen because its absorption maximum nearly coincides with the maximum of the PBI aggregates and because the formation of supramolecular structures allowing for interactions between excited molecules is not expected.

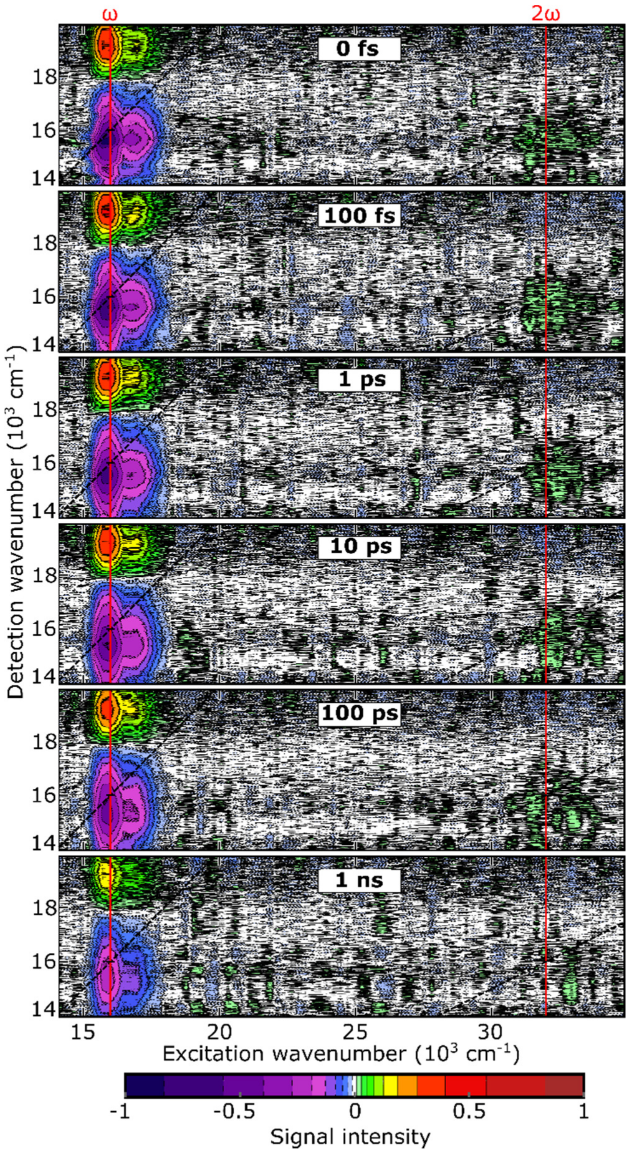

Supplementary Figure 5: EEI2D spectra (real part) of Nile blue at various population times for 34 nJ. Contours are drawn at fractions of 0.01, 0.03, 0.05, 0.08, 0.13, 0.19, 0.27, 0.4, 0.57, and 0.83 of the maximal signal amplitude.

Indeed, the 2D spectra of Nile blue (Supplementary Figure 5) are notably different from the PBI aggregate (Figure 4 and Supplementary Figure 4), although they are measured at identical experimental conditions using high-light excitation intensity (34 nJ). There is only a weak EEI2D signal present in the region of the double excitation frequency. In contrast to MEH-PBI aggregates it does not exhibit any rise with population time that could be attributed to the diffusion-allowed exciton–exciton annihilation. Instead, the signal time evolution "copies" the decay of the absorptive 2D signal. It has been reported previously[13] that Nile blue can be

modelled as a three-level system with approximately equidistantly spaced energy gaps. The observed EEI2D signal thus can be attributed to the direct double excitation of some of the dye molecules.

Supplementary Methods

Number of Absorbed Photons per Monomer

The number of absorbed photons per monomeric PBI molecule is essential for determination of most quantitative values presented in this paper. Its value is determined from the measured spectral absorbance $A(\lambda_{\text{light}})$ of the sample (acquired by spectrometer V-670, Jasco), the spectrum of the excitation laser beam $I_{\text{pump}}(\lambda_{\text{light}})$ (HR 2000+, Ocean Optics), the spatial profiles of the exciting and probing beams $I_{\text{pump}}(x,y)$ and $I_{probe}(x,y)$ (SP 928, Ophir), the total energy of the exciting laser pulse E_{pump} (Vega with PD300 sensor, Ophir) and the known molar concentration of the sample c as follows:

The number of absorbed photons $\tilde{n}_0$ per unit area is calculated from the overlap of the absorption and the excitation laser spectra as

$$\tilde{n}_0 = \tilde{e}_{\text{pump}} \frac{\int I_{\text{pump}}(\lambda_{\text{light}}) \frac{\lambda_{\text{light}}}{h c_{\text{light}}} \left(1 - 10^{-A(\lambda_{\text{light}})}\right) \mathrm{d}\lambda_{\text{light}}}{\int I_{\text{pump}}(\lambda_{\text{light}}) \mathrm{d}\lambda_{\text{light}}}, \qquad (20)$$

where c_{light} is the speed of light, h the Planck constant and λ_{light} the wavelength. Symbol $\tilde{e}_{\text{pump}}$ denotes an effective spatial excitation energy density. We use the spatial intensity distribution of the probe beam (centered on the pump beam) as a weighting factor for contributions from individual lateral pump profile regions. The effective incidence excitation density is thus given as

$$\tilde{e}_{\text{pump}} = E_{\text{pump}} \frac{\int I_{\text{pump}}(x,y) I_{\text{probe}}(x,y) \mathrm{d}x\mathrm{d}y}{\int I_{\text{pump}}(x,y) \mathrm{d}x\mathrm{d}y \int I_{\text{probe}}(x,y) \mathrm{d}x\mathrm{d}y}. \qquad (21)$$

Finally, the number of absorbed photons per molecule (n_0/N) is expressed as

$$\frac{n_0}{N} = \frac{\tilde{n}_0}{c\, N_A\, d}, \qquad (22)$$

where N_A is the Avogadro number and d the cell thickness.

Error Estimate

The dominant source of experimental error in our calculation is the number of absorbed photons per molecule (22). Repetition of experiments resulted in a spread of the maximal TA signal of about ±13% despite all corrections described in Eqs. (20) and (21) were taken. The theory of error propagation was used to determine the corresponding experimental error of all determined quantities. For all of them the experimental error was then combined with the statistical error of the least-square fit and the result is presented as their standard error in the main paper.

In addition, further known sources of error of unknown quantity are present. We expect that the model tends to overestimate the value of exciton delocalization length since we neglected all higher excited states beyond the exciton model of coupled two-level systems. Moreover, the values of transition dipole moments estimated in (2) are only approximate.

Further, the excitation density in the model is characterized by the parameter n_0/N. In the experiment this value varies across the beam profile and from the cuvette front face to its rear face. In our analysis we used the mean value of this parameter defined by Eqs. (20) and (21), which well characterizes the resulting "mean" third-order signal (e.g., TA or absorptive 2D signal of non-interacting species). However, such a mean value of n_0/N characterizes the mean fifth-order signals only approximately. In order to minimize the uncertainty we set the ratio between the sizes of the pump and the probe beams rather big ($\text{FWHM}_{\text{pump}}$:$\text{FWHM}_{\text{probe}}$ = 4.25) and the optical density of the sample rather low (0.2). Note that this type of uncertainty is common to all previous experiments quantifying the diffusion constant on the basis of the fifth-order signal. [6,7,14,15]

Phase Cycling

We employ phase cycling to suppress unwanted contributions that would be detected simultaneously with the desired EEI2D signal. For this purpose, data are repeatedly acquired with different combinations of electric-field phases of the two individual pump pulses. Several phase-cycling schemes are summarized in Supplementary Table 1. Scheme I is employed in the literature on conventional 2D spectroscopy to suppress linear scattering contributions.[16] Here we introduce Schemes II and III that also suppress scattering but in addition display the EEI2D signal. While Scheme III shows EEI2D exclusively, Scheme II allows us to detect, in just

one experiment, absorptive 2D and EEI2D spectra. These contributions can be easily identified and separated in the resulting 2D spectrum since they appear in different regions of the excitation axis.

All data shown in the paper was acquired using Scheme II. For this scheme the weighting factor of the EEI2D signal is twice the factor of the absorptive 2D signal, which proportionally affects the relative intensities of the contributions appearing in the acquired 2D spectrum around the excitation and the double of the excitation frequency. For this reason the factor *w* of Eq. (17) defining the relative intensity of EEI signals in these two regions is 2 x 1/6 = 1/3 in our measurements.

Supplementary Table 1: Comparison between three different phase-cycling schemes. All schemes consist of four independent signal measurements, each denoted (φ_1, φ_2), acquired with different phases of electric fields φ_1 and φ_2 of the two pump pulses and combined as indicated. Individual schemes enhance and suppress the various types of signal differently. Scheme II is used in this work since it allows for the simultaneous detection of absorptive and EEI signals.

Signal phase signature	Signal type	Signal weight for various phase-cycling schemes		
		Scheme I:[16] (0, 0) - (0, π) - (π, 0) + (π, π)	Scheme II: (0, 0) - (0, π/2) - (π/2, 0) + (π/2, π/2)	Scheme III: (0, 0) - (0, π/2) + (0, π) - (0, 3π/2)
1	Transient absorption, Background	0	0	0
$e^{i\varphi_1}$	Scattering from pulse 1	0	0	0
$e^{i\varphi_2}$	Scattering from pulse 2	0	0	0
$e^{i(-\varphi_1+\varphi_2)}$	Absorptive signal, Interference between scattered pump pulses	4	2	0
$e^{i2(-\varphi_1+\varphi_2)}$	EEI signal	0	4	4
$e^{i3(-\varphi_1+\varphi_2)}$	Three-exciton signal	4	2	0

Supplementary References

1. Kasha, M., Rawls, H. R. H. & Ashraf El-Bayoumi, M. The exciton model in molecular spectroscopy. *Pure Appl. Chem.* **11,** 371–392 (1965).
2. Förster, T. Zwischenmolekulare Energiewanderung und Fluoreszenz. *Ann. Phys.* **437,** 55–75 (1948).
3. Suna, A. Kinematics of exciton-exciton annihilation in molecular crystals. *Phys. Rev. B* **1,** 1716–1739 (1970).
4. Gochanour, C. R., Andersen, H. C. & Fayer, M. D. Electronic excited state transport in solution. *J. Chem. Phys.* **70,** 4254–4271 (1979).
5. Torney, D. C. & McConnell, H. M. Diffusion-limited reactions in one dimension. *J. Phys. Chem.* **87,** 1941–1951 (1983).
6. Engel, E., Leo, K. & Hoffmann, M. Ultrafast relaxation and exciton–exciton annihilation in PTCDA thin films at high excitation densities. *Chem. Phys.* **325,** 170–177 (2006).
7. Marciniak, H., Li, X. Q., Würthner, F. & Lochbrunner, S. One-dimensional exciton diffusion in perylene bisimide aggregates. *J. Phys. Chem. A* **115,** 648–654 (2011).
8. Wolter, S. *et al.* Size-dependent exciton dynamics in one-dimensional perylene bisimide aggregates. *New J. Phys.* **14,** 105027 (2012).
9. Knoester, J. Nonlinear optical susceptibilities of disordered aggregates: A comparison of schemes to account for intermolecular interactions. *Phys. Rev. A* **47,** 2083–2098 (1993).
10. van Burgel, M., Wiersma, D. A. & Duppen, K. The dynamics of one-dimensional excitons in liquids. *J. Chem. Phys.* **102,** 20–33 (1995).
11. Ambrosek, D. *et al.* Photophysical and quantum chemical study on a J-aggregate forming perylene

bisimide monomer. *Phys. Chem. Chem. Phys.* **13,** 17649–17657 (2011).
12. Sung, J., Kim, P., Fimmel, B., Würthner, F. & Kim, D. Direct observation of ultrafast coherent exciton dynamics in helical π-stacks of self-assembled perylene bisimides. *Nat. Commun.* **6,** 8646 (2015).
13. Brixner, T., Mančal, T., Stiopkin, I. V & Fleming, G. R. Phase-stabilized two-dimensional electronic spectroscopy. *J. Chem. Phys.* **121,** 4221–4236 (2004).
14. Fennel, F. & Lochbrunner, S. Exciton–exciton annihilation in a disordered molecular system by direct and multistep Förster transfer. *Phys. Rev. B - Condens. Matter Mater. Phys.* **92,** 1–5 (2015).
15. Lin, J. D. A. *et al.* Systematic study of exciton diffusion length in organic semiconductors by six experimental methods. *Mater. Horiz.* **1,** 280–285 (2014).
16. Hamm, P. & Zanni, M. *Concepts and methods of 2D infrared spectroscopy*. (Cambridge University Press, 2011).
